# A Hot Topic: Cancer Immunotherapy and Natural Killer Cells

**DOI:** 10.3390/ijms23020797

**Published:** 2022-01-12

**Authors:** Tatiana Michel, Markus Ollert, Jacques Zimmer

**Affiliations:** 1 Department of Infection and Immunity, Luxembourg Institute of Health, 29 Rue Henri Koch, L-4354 Esch-sur-Alzette, Luxembourg; tatiana.michel@lih.lu (T.M.); markus.ollert@lih.lu (M.O.); 2 Odense Research Center for Anaphylaxis (ORCA), Department of Dermatology and Allergy Center, Odense University Hospital, University of Southern Denmark, DK-5000 Odense, Denmark

**Keywords:** cancer, natural killer cells, immunotherapy

## Abstract

Despite significant progress in recent years, the therapeutic approach of the multiple different forms of human cancer often remains a challenge. Besides the well-established cancer surgery, radiotherapy and chemotherapy, immunotherapeutic strategies gain more and more attention, and some of them have already been successfully introduced into the clinic. Among these, immunotherapy based on natural killer (NK) cells is considered as one of the most promising options. In the present review, we will expose the different possibilities NK cells offer in this context, compare data about the theoretical background and mechanism(s) of action, report some results of clinical trials and identify several very recent trends. The pharmaceutical industry is quite interested in NK cell immunotherapy, which will benefit the speed of progress in the field.

## 1. Introduction

Natural killer (NK) cell immunotherapy for cancer is currently a very hot topic in oncology and generates considerable interest from the scientific community as well as from the pharmaceutical industry. Consequently, the field is very often reviewed in detail [1,2,3,4,5], and therefore we will not re-describe all aspects of NK cells that are in-depth presented in these papers but provide a general introduction before switching to some of the emerging trends. We will also not discuss in detail the repertoire of activating receptors (AR), inhibitory receptors (IR) and their ligands that together control NK cell functions, because this is regularly and comprehensively presented elsewhere [1,2,3,4,5]. Instead, we will focus on the different ways of using NK cells for cancer immunotherapy, with their advantages, current limits, and constraints. Furthermore, we will refer predominantly to recent papers with a focus on the years 2020 and 2021 to be as up-to-date as possible. The articles discussed were non-exhaustively selected through a Pubmed search with the keywords “natural killer cells” AND “immunotherapy”.

Since the first articles about NK cells in the 1970s [6,7], they have always been proposed as ideally suited for cancer immunotherapy [8]. Indeed, one of the fundamental properties of NK cells is, as their name indicates, the capacity to “naturally” kill tumor target cells (as well as virally infected cells) without prior immunization or activation [9,10]. Furthermore, they can perform antibody-dependent cellular cytotoxicity (ADCC), based on the crosslinking with the target cell via an anti-target antibody bound to the NK cell with its Fc part, recognized by the AR CD16 [9,10]. Finally, NK cells also abundantly produce cytokines, chemokines, and growth factors [9,10].

In human peripheral blood, there are two major NK cell subpopulations, defined by the relative expression of CD16 and the adhesion molecule CD56 (NCAM): CD56^bright^CD16^−^, mostly producing cytokines (up to 10% of total peripheral blood NK cells), and CD56^dim^CD16^bright^, the numerically major and predominantly cytotoxic subset (up to 90% of total peripheral blood NK cells) [11,12]. Four other less well-studied subpopulations have been described [11,13]. Natural killer cell functions are governed by a balance between messages received from inhibitory receptors (IR) and AR. If activating messages are predominant and inhibitory messages missing, which is frequently the case for cancer cells, the target will be killed [1,3,5]. A substantial part of the IR is specific for autologous Human Leukocyte Antigen (HLA) class I molecules which are expressed at normal levels on healthy cells (being in this case spared by NK cells) but downmodulated on cancerous cells, leading to their elimination. These IR are the Killer Immunoglobulin Receptors (KIR; ligand: classical HLA class I molecules) and CD94/NKG2A (ligand: HLA-E) [1,3,5]. The cytotoxic process itself is based on the release (degranulation) of the content of the cytolytic granules of the NK cells, containing the apoptosis-inducing molecules perforin, granzymes [1,3,5] and, in human but not in the mouse, granulysin as an additional effector protein with an activity against tumor cells, but also against bacteria [14].

Recent years have shed new light on this lymphocyte population. Thus, it appeared that tissue-resident NK cells are quite different from their peripheral blood counterparts in terms of phenotype and functional behavior and might even represent tissue-specific lineages [15,16,17]. Furthermore, memory NK cells have been discovered, that classified these cells as part of adaptive immunity, whereas the dogma until then was that NK cells are exclusively innate immune cells [18]. Another important item is NK cell education: before becoming functional and responsive to diseased cells in their environment, NK cells must be educated through the interactions of their IR with the ligands of these IR (for example, CD94/NKG2A must “see” HLA-E on surrounding cells) [19,20]. In the absence of IR or HLA class I molecules, NK cells remain hyporesponsive [19,20].

Although, as previously mentioned, NK cells appear as optimal cancer fighters, in practice things are not that simple. First, many of the data about their anticancer activity stem from in vitro studies based on cancer cell lines [21], which may not necessarily reflect the behavior of a complete tumor and its microenvironment in the patient. Nevertheless, it has also been shown that primary tumor cells can be killed by NK cells [22,23], and there are many reports about the efficiency of NK cells in animal models, especially in xenografts (human cancer cells are implanted into mice or rats, and then the animals are treated with human NK cells) [24,25]. Such studies are the standard in top level research papers. They are of course necessary, important, and insightful, but again, the extent to which they might be extrapolated to the human clinical situation is not always clear.

Natural killer cell immunotherapy comes under different forms: either the patient’s own cells are harnessed in vivo by injected antibodies or comparable constructs (checkpoint inhibitors, killer engagers), or NK cells of autologous or allogeneic source are manipulated in vitro (this might include genetic modifications) or not, and then infused into the patient [1,2,3,4,5]. With both ways, efficient answers have been obtained against hematopoietic cancers, whereas, similar to the obstacles to the chimeric antigen receptor (CAR)-T cell therapy, the tumor microenvironment (TME) in solid malignancies still remains a major problem [26].

## 2. Natural Killer Cell Sources for Immunotherapy

As mentioned above, NK cells may provide from autologous sources, in which case a leukapheresis is performed in the patient with the subsequent isolation of peripheral blood mononuclear cells (PBMC), ideally followed by T and B lymphocyte depletion (immuno-magnetic methods). The enriched NK cell fraction is then stimulated with interleukin (IL)-2, which activates the cytotoxic activity of the effectors. Historically, PBMC were cultured with IL-2 for a few days and then infused into the patient (with renal cell carcinoma or metastatic melanoma) together with high dose IL-2 that provoked huge side effects such as a serious vascular leak syndrome. The transferred cells were not pure NK cells, but “lymphokine-activated killers” composed of NK and T lymphocytes, and the overall response rate was rather disappointing [1,27,28]. However, at that time, the knowledge about NK cell biology was still in its infancy, and particularly the various IR and AR had not yet been described. Thus, taking into consideration the missing self-concept stipulating the existence of IR specific for autologous HLA class I molecules [29], it is quite likely that at least the cancers that have not lost the expression of these molecules to a significant extent will be resistant to the autologous NK cells, even if the latter are activated [1,3]. In addition, patients’ NK cells might have suffered from prior treatment options [3]. It was later also found out that the NK cells were in competition with T regulatory cells (Treg) for the infused IL-2; the latter cell population being advantaged because of the expression of the high affinity α chain of the IL-2 receptor, called CD25 [30]. However, in most instances the NK cells are strongly pre-activated and administered together with or following additional treatment modalities [1].

Allogeneic or haploidentical cell sources are another, nowadays most frequently chosen option for adoptive NK cell therapy [1,3,5]. The cells can be obtained from peripheral blood, from umbilical cord blood (usually containing a higher percentage of NK cells), or from the placenta [1,3,5]. After in vitro activation and expansion, the T cell-free NK cell products are infused into the patients. It is crucial to carefully eliminate as much as possible residual allogeneic T cells because of the risk of graft versus host disease (GvHD) that is mediated by the latter but not by NK cells in principle [1,31]. In addition, patients receive a lympho-depleting but not myelo-ablative chemotherapy before NK cell transfer to create a favorable environment for transient engraftment [1,3,31].

A list of selected clinical trials based on the infusion of variably activated and expanded, but genetically not modified NK cells is presented in reference [1].

Another relatively easily expandable cell source are NK cell lines, such as NK-92, derived from a patient with a large granular lymphocyte (LGL) lymphoma. The latter is at present the only NK cell line used for human immunotherapy protocols, as others available (NK-YS, KHYG-1, NKL, NKG, SNK-6, IMC-1, NK3.3) have been shown not to consistently display the same high level of cytotoxic activity [32]. Overall, Klingemann et al. emphasized the advantages of NK-92 over blood NK cells as the therapeutic source, but the corresponding paper [32] dates back to 2016, when several state-of-the-art techniques used nowadays (notably for chimeric antigen receptor (CAR)-NK cells) were still in an earlier stage. A common characteristic of all these NK cell lines is their dependency on IL-2; however, the development by nonviral transfection of the NK-92MI derivative, which produces its own IL-2 but retains most properties of the parental cells, circumvents this problem [33]. It is an advantage that NK-92 cells do not express KIR but only NKG2A as HLA class I-specific IR, they are CD16- at baseline and therefore cannot mediate ADCC, which is one main mechanism of action of anti-tumor monoclonal antibodies [32]. NK-92 cells transfected with a high affinity human CD16 have been generated to enlarge the functional possibilities of the cell line [34,35]. These cells are available from the American Type Culture Collection (ATCC) [35].

In an interesting proof-of-concept study of an affinity-optimized, second generation CD38-targeting CAR (see later for more details about CAR-NK cells) with a costimulatory CD28 domain, retrovirally transduced into the KHYG-1 NK cell line, Stikwoort et al. observe an intense killing of CD38^high^ multiple myeloma (MM) cell lines and primary cells, whereas nonmalignant hematopoietic cells with low or absent CD38 expression are spared. The cytotoxic activity even extends to MM cells resistant to the anti-CD38 antibody daratumumab [36]. Thus, an anti-CD38 CAR-NK therapy could be a good option for an off-the-shelf fight against MM, be the cellular support KHYG-1 or NK-92. Like the original NK-92 line, KHYG-1 cells are CD16- [32].

Yet another example for the expanding number of NK cell lines examined for their therapeutic potential is NK3.3, a unique IL-2-dependent clonal line obtained in the 1980s from the peripheral blood of a healthy donor by the Kornbluth lab [37]. In this case, the focus of interest is actually not the cell line itself, but the extracellular vesicles it is releasing. They have the classical NK cell extracellular vesicle content with cytotoxic molecules [38,39] and several miRNA and efficiently lyse a small panel of hematopoietic and breast cancer cell lines, while normal peripheral blood lymphocytes are resistant [38]. This cell line could, in case the extracellular vesicles hold their promise, become a privileged off-the-shelf supplier of these subcellular fragments, which might be more advantageous and homogeneous than vesicles from a polyclonal bulk NK cell population [38].

However, a detailed investigation by Gunesh et al. [40] of the genomic, phenotypic, and functional profiles of several NK cell lines, among them NK-92 and NK3.3, revealed important differences, and this incites to carefully study the properties of a given line before using it for research and even more for administration to human patients.

A clear advantage of NK cell lines is that they can proliferate indefinitely and do not need to be stimulated before adoptive transfer, except for the IL-2 supply that can be circumvented with NK-92MI. A potential problem is their malignant status, so that they have to be lethally irradiated beforehand. This, in turn, has a negative impact on their in vivo persistence, with the frequent necessity of several infusions.

Further NK cell sources can be CD34+ hematopoietic stem cells (HSC) that are first cultured with a cytokine cocktail to differentiate them into NK cells, and then the latter are expanded in vitro before administration to the patient. Likewise, adult induced pluripotent stem cells (iPSC) derived from skin fibroblasts or PBMC, can be put in culture with growth factors favoring a hematopoietic differentiation first, then with the NK cell-inducing cytokine mixture, and finally with feeder cells allowing their dramatic proliferation [1,3].

## 3. Methods for the Massive Expansion of NK Cells for Immunotherapy

The mere culture of donor-derived NK cells in IL-2 with or without other cytokines (such as IL-15) efficiently activates their cytotoxic activity but does not induce a sufficient proliferation and expansion [1], except in the case of the addition of the anti-CD3 antibody OKT3, which allows an expansion factor of on average 1600 within 20 days in MM patients [41]. Natural killer cell lines (NK-92MI and KHYG-1) can be expanded to the numbers needed by culture without (NK-92MI) or with (KHYG-1) exogenous IL-2 and do not, in principle, need feeder cells to proliferate.

Several other methods based on the co-culture of PBMC with feeder cells have been described: (i) PBMC with irradiated cells of the Wilms tumor line HFWT (expansion between 58 and 401 fold depending on the duration of the culture, which was 10–21 days, and ended up in approximately 70% of activated NK cells) [42,43], (ii) purified NK cells cultured with autologous PBMC [44,45] with an expansion up to 2500 fold at day 17 [44], and (iii) culture of PBMC with irradiated Epstein-Barr virus-transformed B lymphoblastoid cells [46] or with the Burkitt lymphoma Daudi [47] at a PBMC:B cell ratio of 5:1 in the presence of 100 UI/mL of exogenous IL-2. In this case, the proliferation starts at day 6 after a restimulation with the feeder cells and is then impressive until day 10–day 12. The resulting NK cells are largely predominant over T lymphocytes in the cultures and highly activated [46,47]. This method works in principle with all types of B lymphoblastoid cell lines, but the NK cell yield is somewhat higher when the feeder cells are devoid of HLA class I molecules.

This might be reminiscent of the fact that K562 (a HLA class I- chronic myeloid leukemia cell line in blast crisis) was subsequently shown to support a massive expansion of NK cells (median 376 fold [1]) when transduced with membrane IL-15 and 4-1BBL, the ligand for the NK cell AR CD137 [1,48]. Of course, although these conditions are not predominantly favorable for T cell expansion, the latter must be depleted either before or after the NK cell cultures. This system has been adapted for Good Manufacturing Practice (GMP) situations. Although the K562 cells are irradiated and in addition in principle killed by the NK cells, it has to be carefully checked that none of them remain in the final product before adoptive transfer into patients [1,48,49].

The team of Dean A. Lee further improved the method by using the K562 cell line expressing membrane IL-21 and 4-1BBL. With this approach, a mean NK cell expansion of 47.967 fold was obtained compared to 825 fold with the membrane IL-15 variant [50,51]. Interestingly, there was no sign of senescence even after six weeks, but on the contrary, an increase in the length of telomeres [3,51]. As apparently this line can now be only used in one single center, Ojo et al. developed a new feeder cell line called ‘NKF’, and consisting in the myeloid leukemia cells OCI/AML3 expressing membrane IL-21 [52]. The latter supports a strong expansion (more than 10.000 fold) of highly active NK cells over five weeks [52].

Other authors actively tried to further optimize the NK cell expansion protocols. Thus, Thangaraj et al. [53] cultured PBMC with a K562–OX40L–membrane IL-18–membrane IL-21 feeder cell line in the presence of soluble IL-2/IL-15, and observed a 9.860 fold increase in NK cell numbers from healthy donors versus 4.929 fold from multiple myeloma patients, in which NK cells are usually dysfunctional, after a culture period of four weeks [53]. These NK cells (over 80% purity) were highly cytotoxic to the three tested tumor cell lines and upregulated the most important AR [53].

Min et al. likewise demonstrated a significant NK cell expansion out of T cell-depleted PBMC stimulated with the T cell lymphoma cell line Hut 78 transduced with various activating molecules, the combination 4-1BBL–membrane tumor necrosis factor (TNF)-*α*–membrane IL-21 being the most efficient [54].

The starting material for such expansion endeavors can be PBMC, CD34+ HSC, or appropriately differentiated iPSC. It is suggested to prefer haploidentical (or allogeneic) NK cells rather than autologous ones, especially to avoid their inhibition by self HLA class I molecules interacting with specific IR, as mentioned above.

The usefulness of allogeneic NK cells has been clearly demonstrated by the Velardi group in the early 21st century, when they performed haploidentical T cell-depleted HSC grafts into acute myeloid leukemia (AML) patients [55]. In these recipients, almost no GvHD and no relapses were observed, and based on mouse studies done in parallel, it was assumed that the allogeneic NK cells killed residual leukemic cells (graft versus leukemia—GvL), as well as recipient T cells and dendritic cells, so that no graft rejection took place. More mechanistically, those donor NK cells not expressing a KIR recognizing a HLA class I molecule of the recipient were not inhibited by the recipients’ cells and lysed them (KIR mismatch in the donor to recipient direction). This looks fantastic, but the same therapeutic approach had almost no effect in acute lymphoblastic leukemia (ALL) individuals [55], as ALL blasts might be inherently more resistant to NK cells due to a lack of ligands for AR. Several subsequent studies, mostly by the same team, confirmed this beneficial allogeneic NK cell effect in AML. However, a very recent prospective study, in which more than one third of the patients surprisingly received an un-manipulated, non-T cell-depleted graft, came to more moderate conclusions [56]. Natural killer cell alloreactivity was still of interest, but only in the patients having received a graft depleted in T cells, where it was related to a reduced incidence of acute and chronic GvHD. Overall, the cohort was very heterogeneous and contained a high number of individuals with ALL, and an astonishing high percentage (20–25%) of GvHD in the T cell-depleted group. The authors speculate that in these patients, the necessary immunosuppressive treatment of GvHD might have blunted NK cell alloreactivity [56]. A previous report had already described an acute GvHD in five out of nine subjects after a HLA-matched unrelated transplantation of peripheral blood stem cells together with donor-derived activated NK cell infusions (stimulation with the artificial antigen-presenting cell line KT32.A2.41BBL.64, a lentivirus-transduced variant of K562, plus recombinant human IL-15; reason for the graft: high-risk solid tumors in children and adults) [57]. Three of the patients had severe (grade 4) acute GvHD. Although the stem cell product and the NK cells were T cell-depleted, and the remaining number of T cells was very low (in the 10^3^–10^4^ range), the authors concluded that the highly activated infused NK cells participate in GvHD pathogenesis, perhaps by favoring T cell alloreactivity [57].

Along the same lines, whereas most reviews and original research papers claim that NK cell immunotherapy is safe and well tolerated, Mamo et al. retrospectively analyzed infusion reactions in 130 cancer patients from nine different clinical trials from the same institution [58]. The patients had recurrent solid cancer in some trials and relapsed/refractory hematological cancer in the remaining ones and all had received salvage chemotherapy that had failed. The allogeneic NK cell preparations were CD3-depleted, most of them also CD19-depleted, and all were activated prior to infusion with IL-2 or IL-15 overnight. The cytokines were then carefully washed out. Side effects were recorded from the time of NK cell infusion until four hours later. Four hours after the NK cell administration, the patients were started on subcutaneous IL-2 (seven trials) or IL-15 (once subcutaneous and once intravenous).

A total of 91% of patients showed infusion reactions, the most frequent ones being chills, hypertension, fever, and headache. However, the side effects were most often of minor intensity (grade 1 or 2), although 28% of the individuals experienced grade 3 reactions and one a grade 4 life threatening hypotension. No death occurred. With an overall response rate of 27.6%, no difference in this regard could be observed between those with severe and those with minor infusion reactions. Hematological cancers responded better than solid tumors [58].

An important point here is that there was no association between the infused NK cell dose and the occurrence of grade 3 side effects, leading the authors to conclude that possibly the NK cells themselves are not responsible. Indeed, they found that the content of monocytes in the infusion product was correlated with headache and with high-grade cardiovascular effects [58]. This might be an important observation, given the requirement of monocytes and their cytokines, such as IL-1 and IL-6, for the occurrence of CRS and neurotoxicity during CAR T cell therapy [59].

Although these different clinical situations are not exactly the same, it should be kept in mind that NK cells, even if probably mostly beneficial, may provoke serious adverse events and off-target effects, and must be handled with care similarly to all other interventions on the immune system [60,61].

## 4. Adoptive Transfer of NK Cells

Two major possibilities for taking advantage of the anti-tumor properties of NK cells exist: (i) adoptive transfer, where the cells are expanded and activated in vitro and then (re-) infused into the patient, and (ii) harnessing the patient’s own NK cells to fight their tumor through the administration of checkpoint inhibitors, monoclonal tumor-specific antibodies or bi-and trispecific killer engagers.

Regarding the first option, we have already addressed some aspects above.

Silla et al. recently published a proof-of-concept paper about a clinical trial in which 13 patients (14 treatment courses) with relapsed/refractory AML received several infusions of K562/IL-21-expanded CD56^bright^/CD16^bright^ activated NK cells, with an overall response (OR) of 78.6% and complete remissions (CR) in 50% of the courses [62]. Regarding adverse events, many were registered, but most of them could not be attributed to the NK cells. However, one case of GvHD and one grade 4 central nervous system toxicity were observed, the latter being in fact an on-target anti-leukemic effect that resolved. No dose-limiting toxicity and no cytokine release syndrome occurred [62].

Among the different imaginable approaches, one that seems particularly promising is the administration of allogeneic cytokine-induced memory-like NK cells (CIML) [63]. They are prepared in vitro by 12–16 h exposition to the cytokines IL-12, IL-15, and IL-18, which confers them a stronger activity and a better response to stimulating signals that persist for weeks to months if low-dose IL-2 is provided [3,63]. In a phase I clinical trial with a total of 15 evaluable patients with relapsed/refractory AML, the infusion of such cells was followed by three CR and four complete remissions with incomplete neutrophil recovery (CRi), which is quite a remarkable result. No major toxicity, particularly no cytokine release syndrome, no GvHD, and no neurotoxicity were observed. These memory-like NK cells expanded in vivo in the AML recipients and their presence was demonstrated for several weeks. They displayed a phenotype different from control NK cells (baseline in vitro incubation with the cytokine cocktail) and improved expression of several important AR as well as activation markers, such as CD25, CD69, and CD137 [63]. In contrast, a high expression level of the IR NKG2A was associated with treatment failure.

The logical next step, besides ameliorating and extending the efficiency in hematological malignancies, namely to test this treatment modality in solid tumors, was likewise addressed by the Fehniger group [64]. In this field, advanced melanoma is an example with a highly unmet clinical need. When performing in vitro experiments and using the power of mass cytometry, the authors could show that the CIML NK cells had strong cytolytic activity and cytokine production towards allogeneic and autologous melanoma target cells, suggesting that this type of NK cell effectors can overcome the frequently observed dysfunction in melanoma patients. In a mouse xenograft model, it appeared that the CIML NK cells had a better efficiency than conventional NK lymphocytes against transplanted melanoma tumors [64].

More detailed reviews specifically about CIML and adoptive NK cell transfer in melanoma and its different advantages and pitfalls are provided by Mikelez-Alonso et al. [65] and van Vliet et al. [66], respectively.

Another interesting option is represented by the so-called adaptive NK cells, which proliferate in response to the interaction of the complex formed between the non-classical HLA class I molecule HLA-E and a peptide derived from the human cytomegalovirus-encoded (HCMV) protein UL40, and the NK cell AR NKG2C [3]. Therefore, they are also a type of memory-like NK cells. This subset is phenotypically characterized by the presence of NKG2C and CD57 (terminal differentiation marker), as well as CD2, autologous HLA class I-specific KIR, ILT2 (or CD85j, a broad spectrum HLA class I-specific IR), and the anti-apoptotic molecule Bcl-2, and are epigenetically remodeled. In contrast, NKG2A, the natural cytotoxicity receptors (NKp30, NKp44, NKp46), the transcription factor PLZF, FcεRIγ, and the enzyme Syk are down-modulated [67,68]. The adaptive NK cells need three stimuli to emerge: (i) the presentation of the appropriate viral ligand bound to HLA-E to the AR NKG2C, a co-stimulation (particularly via CD2), and the presence of pro-inflammatory cytokines, such as for example IL-12. The resulting NK cells are heterogeneous in phenotype, epigenetic modulation, and functional behavior [67]. However, they share strong and efficient effector functions such as a proficient ability to perform ADCC, high cytokine production and a resistance to myeloid-derived suppressor cells [69] and T regulatory cells [70], all of which render them interesting and important in viral infections, transplantation, vaccination, and obviously cancer immunotherapy [67].

Initially exclusively observed in HCMV seropositive donors, Hammer et al. [71] also obtained adaptive NK cells from HCMV-negative individuals when the three activating parameters mentioned above were present. In addition, they determined a certain hierarchy in the CMV peptides’ ability to induce the adaptive NK cells.

As adaptive NK cells are terminally differentiated, it is more difficult to expand this subset than their conventional (or canonical) counterparts. Nevertheless, Liu et al., by culturing PBMC with the HLA class I- B lymphoblastoid cell line 721.221 transfected with HLA-E in the presence of exogenous IL-15, observed a quite selective, 2.4 fold expansion of NKG2C+ NK cells expressing a single self-specific KIR, which makes them an interesting product for allogeneic NK cell therapy [72]. These cells were highly effective against acute lymphoblastic leukemia blasts. Other authors described the use of the GSK3 inhibitor CHIR99021 together with IL-15 to induce a feeder cell-free adaptive NK cell proliferation, with some preclinical success and ongoing clinical trials [3,67,73]. Nevertheless, there is most likely still room for improvement for generating enough of these highly potent effectors.

In this context, experiments as well as clinical trials are underway to promote the differentiation of adaptive NK cells, starting from iPSC [3].

Induced pluripotent stem cell-derived NK cells might also be better suited than primary peripheral blood lymphocytes for the generation of CAR-NK cells, which can likewise be produced from inherently cytotoxic cell lines such as NK-92 or from cord blood. These CAR-NK cells are a potent approach to specifically focus on a tumor antigen on the cancer cells and to therefore endow the NK cells with the same specificity than CAR-T cells. It was frequently objected that NK cells are never antigen-specific, an argument that is no longer valid with the arrival of CAR-NK cells in preclinical studies and clinical trials. Adult iPSC allow to obtain high numbers of NK cells, are easily accessible (theoretically, any somatic cell type might be reprogrammable into iPSC) because mostly skin fibroblasts or PBMC are used [74,75], and avoid the ethically highly debatable approach with embryonic stem cells. Once suitable homogeneous iPSC clones are selected, they can be banked and are further expandable and differentiable into the desired end product. Several methods and protocols to end up with NK cells have been published, although they are not yet entirely problem-free [74,75].

Most currently constructed CAR for NK cells are constituted of (i) a single chain variable fragment (scFv, a small part of the specific variable domain of an antibody) directed towards an antigen expressed as selectively as possible by the targeted tumor, (ii) an intracellular tail which consists in an activating molecule such as CD3*ζ*, and (iii) one or more co-stimulatory moieties (for example, 4-1BB or 2B4) [74] A hinge region and a transmembrane domain complete the construct [76]. In order to increase the in vivo persistence of the NK cells, it is possible to include IL-15 for a constitutive expression [76,77]. The legendary resistance of NK cells to transfection can be overcome with retroviral- or lentiviral transduction, which in turn might represent a problem if the virus integrates into the NK cell genomes. Recent work is based on alpharetroviral vectors with a more favorable pattern in this regard [76,78].

In a landmark study published in 2020, Liu et al. reported on a phase I-II clinical trial based on CAR-NK cells with a CAR composed of an anti-CD19 scFv, a CD28 costimulatory sequence and a CD28.CD3*ζ* signal transducing element, together with the human IL-15 gene and an inducible caspase 9 to get selectively rid of the CAR-NK cells in case of major adverse events (the latter, however, did not happen during the clinical trial). The construct was retrovirally transduced into cord blood-derived NK cells expanded with the K562 cell line expressing membrane IL-21 and 4-1BBL in the presence of IL-2 [77]. Then, the CAR-NK cells were administered to heavily pretreated patients with relapsed/refractory CD19-expressing hematologic malignancies (chronic lymphocytic leukemia and B cell lymphomas). The adoptively transferred cells persisted in vivo for a long time, probably due to the continuous presence of IL-15. However, as the donor cells were allogeneic compared to the recipients, the question remains open as to why the recipients did not reject the CAR-NK cells earlier. This might be due, according to the authors, to the presence of IL-15 and the lymphodepleting chemotherapy that preceded the CAR-NK cell transfer. This chemotherapy was also considered responsible for the sometimes severe but reversible myeloid toxicity, whereas no cytokine release syndromes, no severe neurological adverse events and no GvHD were observed [77]. In terms of efficiency, 73% (eight of the eleven treated patients) had an objective response, and among these, seven a CR [77]. Importantly, the CAR-NK cell products were individually prepared for each patient, so that this clinical trial did not yet come up with an off-the-shelf treatment, impatiently expected by the field to be able to include a maximum number of patients.

These CAR-NK cells could in principle be directed against any tumor cell antigen as long as normal tissues are not extensively affected. Thus, a large number of CAR-NK cell clinical trials are currently recruiting and target hematopoietic as well as solid tumors [3,26,79,80]. We have already mentioned the multiple myeloma paper by Stikwoort et al. [36], but there are likewise many other examples of preclinical investigations, such as the work by Lin et al., who constructed a CAR containing the NK cell AR NKp30 (also called natural cytotoxicity receptor 3 or NCR3) targeting one of its ligands, namely B7-H6, expressed by anti-estrogen resistant breast cancer cells. The NK-92 cell line was transduced with the CAR construct and efficiently killed estrogen-resistant MCF-7 breast cancer cell line variants in vitro [81]. Hintz et al. used the NK-92MI derivative to test its efficiency against a prostate cancer cell line and in parallel a prostate stromal cell line (to mimic in vitro the targeting of the tumor and the TME in parallel) [82]. To do so, they transduced the NK cell line with full-length CD64, a high affinity Fc*γ* receptor physiologically expressed only by myeloid cells. Knowing that NK-92MI cells are CD16-, they observed a high level of ADCC against both prostate cell lines in the presence of appropriated antibodies. The data was confirmed in an in vivo xenograft mouse model [82], so that with this approach, immunotherapy of prostate cancer and other solid tumors could become possible. The results also emphasize the benefits of including the TME into the therapeutic strategy.

## 5. Harnessing of Autologous NK Cells

A completely different approach is to act on the patient’s own NK cells and to stimulate them via the administration of therapeutic antibodies or NK cell engagers that crosslink the NK cells with the tumor cells and either relieve inhibition by masking exhaustion molecules (immune checkpoints) or triggering AR. Indeed, NK cells in cancer patients are often functionally deficient, and even if it takes less time to generate allogeneic NK cells than autologous T cells in sufficient numbers for an immunotherapy, the tumor might evolve so fast that the patient cannot wait and needs an off-the-shelf treatment [83] which is, as mentioned above, theoretically possible for adoptive NK cell transfer but not yet routinely present in the clinic.

In contrast, monoclonal humanized therapeutic antibodies are available in large numbers on the market, while many others are still in the development phase. The so-called checkpoint inhibitors, such as the anti-CTLA4 antibody ipilimumab [84] or the anti-programmed death-1 (PD-1) antibodies nivolumab and pembrolizumab [85], mostly target exhausted autologous T cells, although NK cells can also express PD-1 [83]. Furthermore, the anti-CD20 antibody rituximab (against malignant B cell neoplasms) [86], as well as the anti-CD38 antibody daratumumab [87] and the anti-CD319 (SLAMF7) antibody elotuzumab [88], which are both part of the treatment arsenal against multiple myeloma, bind to the AR CD16 via their constant Fc portion and to the tumor cells by their variable part specific for the cited tumor antigens. In addition, elotuzumab is also able to activate NK cells through the binding to SLAMF7 expressed by NK cells and thus acts both in a CD16-dependent and -independent manner [88]. These three molecules are already in clinical use and have proven to be quite efficient. However, due to a polymorphism in CD16, patients with a low affinity variant respond less well to monoclonal antibodies [83].

Whereas initially, exhausted T cells were almost exclusively in the focus of checkpoint inhibition strategies, the same principle actually holds true for NK cells from individuals affected by cancer: these lymphocytes are phenotypically abnormal and functionally deficient [64], but it is possible to act on the checkpoints to restore the ability of fighting the tumor. The first monoclonal antibodies targeting NK cell IR and to be clinically tested were the anti-KIR2DL1, -KIR2DL2, and -KIR2DL3 molecule lirilumab, which blocks the inhibitory interaction of the KIR with HLA-C proteins [89], and the anti-NKG2A antibody monalizumab, interfering with the binding of the IR NKG2A, expressed by subsets of NK cells and CD8+ T lymphocytes, with its ligand HLA-E [90,91]. The former was recently tested in clinical trials in combination with the anti-PD-1 antibody nivolumab against hematologic [92] and solid (head and neck) tumors [93] and showed some efficiency, even in terms of disease free and overall survival [93]. Regarding monalizumab, a phase II trial revealed, in combination with the anti-epithelial growth factor receptor (EGFR) antibody cetuximab, an objective response rate of 31% in pretreated head and neck squamous cell carcinoma with tolerable side effects [90]. In contrast, monalizumab monotherapy in recurrent metastatic squamous cell carcinoma of the head and neck was much less efficient, with no objective response and a stable disease in only 23% of the patients [94]. However, overexpression of NKG2A on NK cells from the TME as well as of HLA-E on cancer cells [94] have been described in this neoplasm. This discrepancy between the successful poly-therapy and the moderately efficient monotherapy illustrates well that it seems always better to attack the tumor from multiple sites and through different pathways, and the authors of reports on clinical trials usually insist on the need for such a multiple target approach [90,94].

Further NK cell co-inhibitory immune checkpoints also expressed by other immune cell types that are currently in the focus of interest are T cell immunoglobulin and mucin domain molecule 3 (Tim-3) [95], T cell immune receptor with immunoglobulin and ITIM domains (TIGIT) [96], and CD112 receptor (CD112R) [97]. Whereas Tim-3 recognizes galectin-9, HMGB1 and CEACAM1, the latter two are specific for the nectin family molecules CD112, CD113, and CD155 [98], as well as for the recently detected nectin-4 in the case of TIGIT only [96]. Nectins are frequently up-regulated on cancer cells. They are also the ligands for the AR DNAM-1 (CD226) [98].

Several phase I and/or II clinical trials targeting the PD-1/PD-L1 pathway together with Tim-3 in solid tumors are currently ongoing [95]. Although the aim is to relieve T cell exhaustion, the common expression of the markers PD-1 and Tim-3 by NK cells most likely also can be expected to stimulate these lymphocytes. Clinicians use either bispecific anti-PD-1/Tim-3 antibodies or humanized monoclonal anti-Tim-3 molecules such as sabatomimab.

Furthermore, a plethora of anti-TIGIT monoclonal antibodies are in advanced clinical development for solid tumors, such as for example domvanalimab or vibostolimab (phase II) and tiragolumab and ociperlimab (phase III) [96]. The mechanism of action of TIGIT is that of a non-MHC class I-specific IR that impairs NK cell functions upon binding to its ligand. In addition, it competes with DNAM-1 for the ligation to CD155 [96].

The expression of TIGIT relative to the six different peripheral blood NK cell subsets from healthy donors was recently investigated by Esen et al. [99], who interestingly found that this receptor was present at the highest level on CD56^dim^CD16^dim^ NK cells [13].

Regarding CD112R, it is a molecule considered as having a high potential in cancer immunotherapy and it has already been shown that its ligand CD112 has a diagnostic and prognostic value in several cancers of the digestive tract and others [97].

Instead of being based on entire monoclonal antibodies that have quite a heavy molecular weight, the bi- and tri-specific killer engagers (BiKE and TriKE) are composed of two or three single chain variable antibody fragments (scFv) specific for NK cell AR and tumor antigens, respectively [3,100]. They crosslink NK and tumor cells and induce the degranulation of the cytotoxic content of the NK cells, leading to the lysis of the cancer. In contrast to monoclonal antibodies that can mediate ADCC if their Fc constant part is not modified to avoid this binding, BiKE and TriKE do not act via ADCC but perform a redirected killing. To stimulate autologous NK cells and increase their survival, the NK-activating cytokine IL-15 is now frequently integrated into the molecules. For example, the 161519 TriKE is directed against the AR CD16 on NK cells, towards the B cell marker CD19, highly expressed on B cell malignancies, and contains IL-15 for additional NK cell activation [100,101]. A second generation TriKE (“161533”) directed at the myeloid leukemia cell marker CD33 shows improved characteristics in vitro [102]. Several clinical trials with BiKE and TriKE are ongoing [3,103].

The same concept, more or less, is used for the tetravalent bispecific NK cell engager AFM13, composed of two anti-CD16 and two anti-CD30 moieties [104]. It is currently in clinical phase II development against certain hematologic malignancies. Other comparable constructs are likewise tested in phases I or II [103,105]. They function according to the principle of redirected killing.

A slightly different approach has been taken by the Vivier group who developed several so-called natural killer cell engagers (NKCE) [103,106]. The most accomplished form is composed of an anti-NKp46 Fab (variable fraction of an antibody), a Fc fragment binding to CD16, and an anti-tumor antigen Fab. It mediates a very strong NK cell activation, superior to bispecific compounds and therapeutic antibodies acting through ADCC [106]. This molecule is part of a versatile platform where other Fab and engineered Fc fragments can be introduced to either increase or reduce the interaction with the Fc*γ* receptor CD16 [103].

A potential drawback of targeting CD16 is the rapid downmodulation of this AR after interaction with ligands [103]. This might be circumvented by the use of inhibitors of the metalloprotease ADAM17 [107], which is responsible for the cleavage, or by the involvement, together with NKp46, of RA other than CD16.

## 6. Conclusions and Perspectives

**Figure 1:**
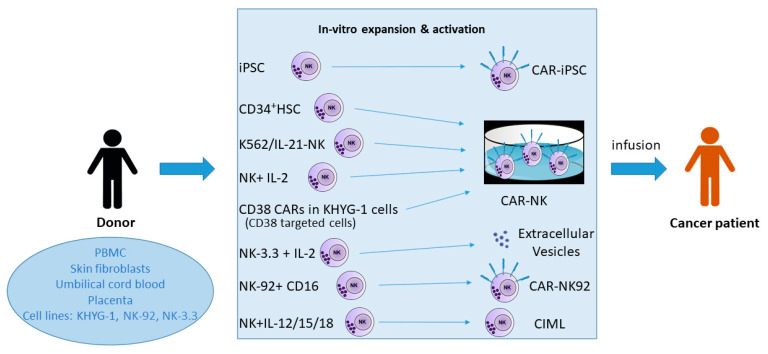
NK cells based cancer immunotherapies. Abbreviations: PBMC: peripheral blood mononuclear cells; iPSC: induced pluripotent stem cells; HSC: hematopoietic stem cells; CAR-NK: chimeric antigen receptor natural killer cell; CAR: chimeric antigen receptor; CIML: cytokine-induced memory-like NK cells.

After this non-exhaustive journey through various options of NK cell-mediated cancer immunotherapy (Figure 1), either already in clinical use or in advanced preclinical stages, the conclusion might be that there is still a long way to go before NK cell products can be used routinely and as off-the-shelf oncologic treatment modalities. Nevertheless, the advantages of NK cells over CAR-T cells are obvious (faster availability, lesser cost, no GvHD, no CRS, no neurotoxicity), although not unanimously recognized by the field. It might of course happen that with an increasing number of NK cell-treated patients, more adverse effects will show up.

As emphasized by many authors interested in immunotherapy, a treatment with just one option might be less efficient than a poly-therapeutic approach attacking the tumor from multiple sites and fighting the cancer itself, but also the immunosuppressive TME that can abolish the effects of all ingenious engineering of immune cells if they are not armed to face this environment, especially in solid tumors.

Further modalities are in preclinical development, whereby the nanotechnologies bear a major hope [65,108,109].

Overall, it might be predicted without too much risk that NK cell immunotherapies, whatever their precise form, are on their way to becoming a major component of the future global oncologic approach to cancer patients.

## Data Availability

Not applicable.
